# Crystal structure of rubidium peroxide ammonia disolvate

**DOI:** 10.1107/S2056989017000354

**Published:** 2017-01-17

**Authors:** Tobias Grassl, Nikolaus Korber

**Affiliations:** a Institut für Anorganische Chemie, Universität Regensburg, Universitätsstrasse 31, 93053 Regensburg, Germany

**Keywords:** crystal structure, rubidium, peroxide, ammonia disolvate

## Abstract

The title compound, Rb_2_O_2_·2NH_3_, has been obtained as a reaction product of rubidium metal dissolved in liquid ammonia and glucuronic acid. As a result of the low-temperature crystallization, a disolvate was formed. To our knowledge, only one other solvate of an alkali metal peroxide is known: Na_2_O_2_·8H_2_O has been reported by Grehl *et al.* [*Acta Cryst.* (1995), C**51**, 1038–1040]. We determined the peroxide bond length to be 1.530 (11) Å, which is in accordance with the length reported by Bremm & Jansen [*Z. Anorg. Allg. Chem.* (1992), **610**, 64–66]. One of the ammonia solvate molecules is disordered relative to a mirror plane, with 0.5 occupancy for the corresponding nitrogen atom.

## Abstract 2

The title compound, Rb_2_O_2_·2NH_3_, has been obtained as a reaction product of rubidium metal dissolved in liquid ammonia and glucuronic acid. As a result of the low-temperature crystallization, a disolvate was formed. The peroxide bond length is 1.530 (11) Å

## Chemical context   

The crystal structure of the title compound was determined in the course of investigations regarding the reactivity of carbohydrates towards alkali metals and NH_3_ in solutions where liquid ammonia itself is used as solvent. The source of the peroxide anion could not be explicitly traced back but it seems to have its origin in oxygen gas from intruding atmosphere due to undetected leakage in the reaction vessel.

## Structural commentary   

**Figure 1:**
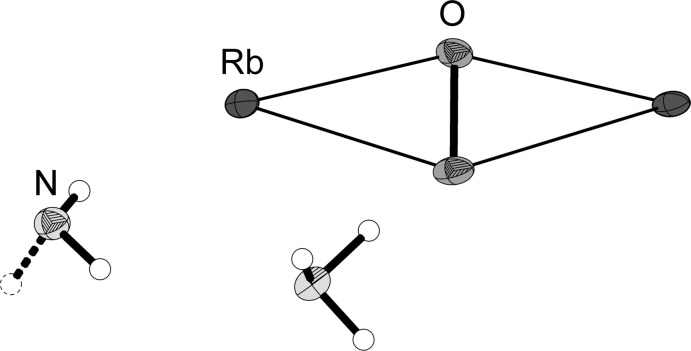
The asymmetric unit of the title compound, with the atom labeling and displacement ellipsoids drawn at the 50% probability level.

**Figure 2:**
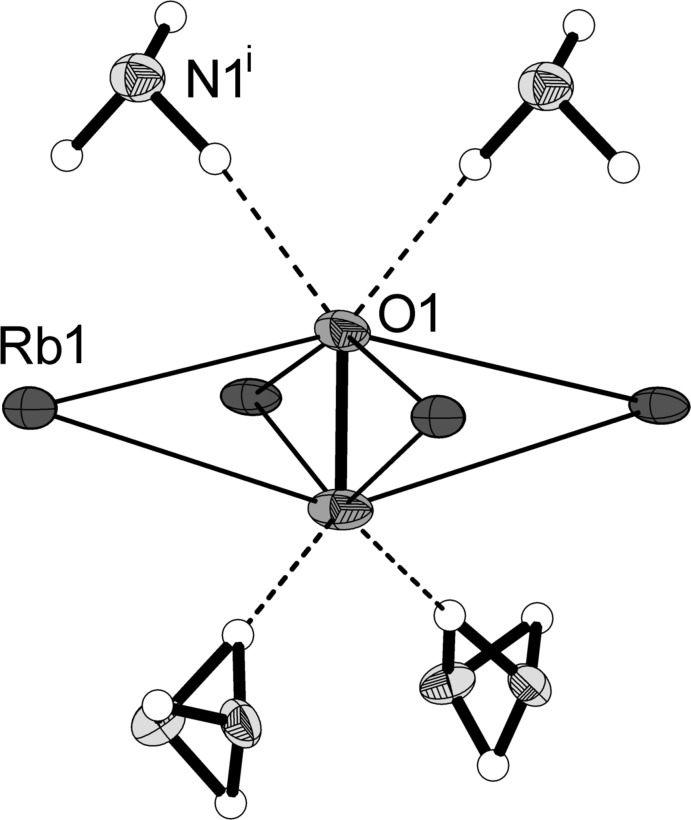
The environment of the peroxide anion. Displacement ellipsoids are drawn at the 50% probability level. [Symmetry code: (i) −1 + *x*, *y*, *z*.]

**Figure 3:**
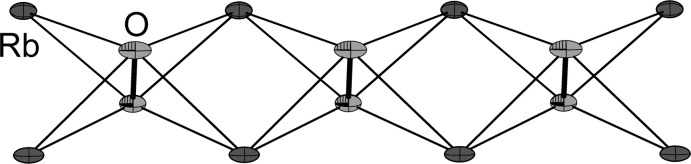
One-dimensional infinite strands formed by peroxide anions and rubidium cations. Displacement ellipsoids are drawn at the 50% probability level.

The asymmetric unit contains one peroxide anion, two charge-compensating rubidium cations and two ammonia mol­ecules (Fig. 1 ▸). Except for one nitrogen atom (N1, showing half-occupancy) and one hydrogen atom (H2*B*), all other atoms are located on mirror planes. The anion is surrounded by four rubidium cations located around the girth of the peroxide ion (Fig. 2 ▸). This unit forms one-dimensional infinite strands by sharing one common edge of a distorted plane of four Rb ions (Fig. 3 ▸). This structural motif can also be observed in potassium acetyl­ide K_2_C_2_ (Hamberger *et al.*, 2012 ▸). The peroxide bond length was determined to be 1.530 (11) Å. The anion–cation contacts range between 2.790 (5) Å and 2.917 (6) Å. The coordination number of the cations is 8.

**Figure 4:**
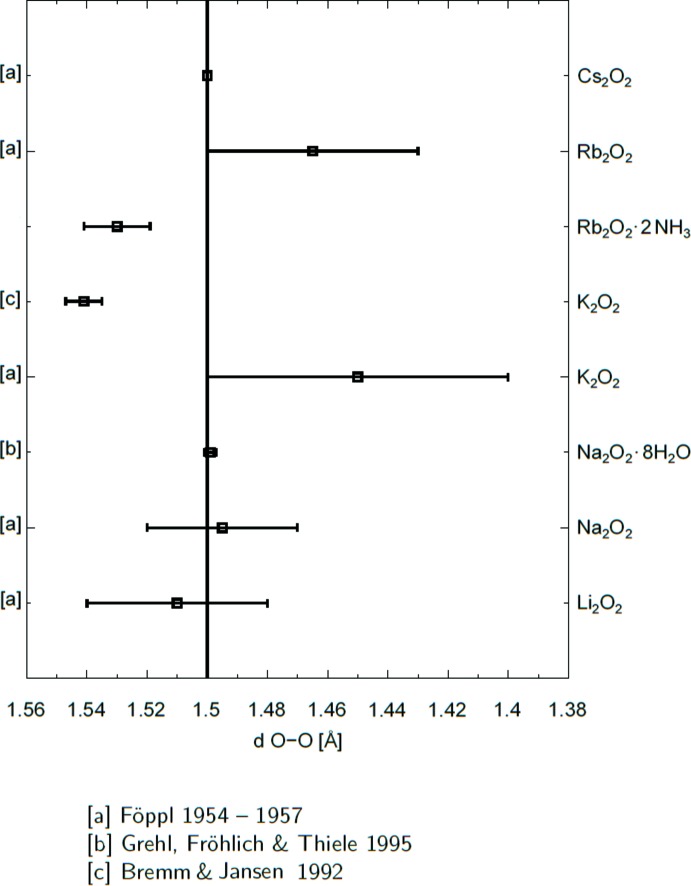
Comparison of peroxide bond lengths in different compounds. The vertical line shows the peroxide bond length commonly used in the literature. Each data point is shown with its standard uncertainties.

The O—O bond length of the peroxide anion is longer than the value found in the literature based on the work of Föppl which is approximately 1.49 Å. In Fig. 4 ▸, a comparative view of bond lengths is presented based on the work of Bremm & Jansen (1992 ▸), Föppl (1954 ▸, 1955 ▸, 1957 ▸) and Grehl *et al.* (1995 ▸).

## Supra­molecular features   

Despite the low ammonia content, numerous hydrogen bonds can be observed and the NH_3_ molecules bridge the peroxide anions. The peroxide anion shows five contacts to ammonia molecules, forming a three-dimensional network in the packing. The distances between donor and acceptor atoms ranges from 2.926(15) Angstrom to 3.597(16) Angstrom, which is commonly observed in ammoniates. Numerical details of the hydrogen-bonding inter­actions are given in Table 1 ▸.

## Synthesis and crystallization   

500 mg (2.58 mmol) d-glucuronic acid and 880 mg (10.29 mmol) rubidium were placed under an argon atmosphere in a reaction vessel and 25 ml of dry liquid ammonia were condensed. The mixture was stored at 237 K for five days. The flask was then stored at 161 K for several months. After that period, clear needle-shaped colorless crystals of the title compound could be found at the wall of the flask.

## Refinement   

Crystal data, data collection and structure refinement details are summarized in Table 2 ▸. The nitro­gen atom N1 is disordered with 0.5 as the site occupation factor. All hydrogen atoms could be located in difference map and and their positions were refined freely with a common *U*_iso_(H) parameter. The isotropic displacement parameters were fixed to 0.025.

## Supplementary Material

Crystal structure: contains datablock(s) I. DOI: 10.1107/S2056989017000354/pj2039sup1.cif

Structure factors: contains datablock(s) I. DOI: 10.1107/S2056989017000354/pj2039Isup2.hkl

CCDC reference: 1526250

Additional supporting information:  crystallographic information; 3D view; checkCIF report

## Figures and Tables

**Table 1:** Hydrogen-bond geometry (Å, °)

*D*—H⋯*A*	*D*—H	H⋯*A*	*D*⋯*A*	*D*—H⋯*A*
N1—H1*A*⋯O2	1.05 (14)	1.98 (15)	2.941 (16)	151 (8)
N1—H1*B*⋯O2^i^	0.97 (13)	2.04 (15)	2.926 (15)	151 (8)
N1—H1*C*⋯O1^ii^	0.82 (14)	3.07 (16)	3.597 (16)	125 (11)
N2—H2*A*⋯N2^iii^	0.74 (16)	3.03 (12)	3.57 (2)	131 (6)
N2—H2*A*⋯N2^iv^	0.74 (16)	3.03 (12)	3.57 (2)	131 (6)
N2—H2*A*⋯N2^v^	0.74 (16)	3.03 (12)	3.57 (2)	131 (6)
N2—H2*B*⋯O1^ii^	1.01 (11)	1.95 (11)	2.955 (10)	173 (8)

Symmetry codes: (i) 
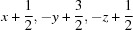
; (ii) 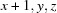; (iii) 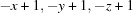; (iv) 
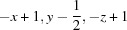
; (v) 
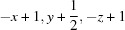
.

**Table 2:** Experimental details

Crystal data	
Chemical formula	Rb_2_O_2_·2NH_3_
*M*_r_	237.01
Crystal system, space group	Orthorhombic, *P* *n* *m* *a*
Temperature (K)	123
*a*, *b*, *c* (Å)	7.3957 (7), 4.0932 (6), 18.1873 (17)
*V* (Å^3^)	550.57 (11)
*Z*	4
Radiation type	Mo *K*α
μ (mm^−1^)	17.66
Crystal size (mm)	0.24 × 0.09 × 0.08

Data collection	
Diffractometer	Agilent SuperNova Dual Source diffractometer with an Eos detector
Absorption correction	Analytical [*CrysAlis PRO* (Agilent, 2012 ▸), based on expressions derived by Clark & Reid (1995 ▸)]
*T*_min_, *T*_max_	0.064, 0.354
No. of measured, independent and observed [*I* > 2σ(*I*)] reflections	2921, 641, 570
*R*_int_	0.057
(sin θ/λ)_max_ (Å^−1^)	0.625

Refinement	
*R*[*F* ^2^ > 2σ(*F* ^2^)], *wR*(*F* ^2^), *S*	0.050, 0.118, 1.35
No. of reflections	641
No. of parameters	51
H-atom treatment	Only H-atom coordinates refined
Δρ_max_, Δρ_min_ (e Å^−3^)	1.12, −1.79

Computer programs: *CrysAlis PRO* (Agilent, 2012 ▸), *olex2.solve* (Bourhis *et al.*, 2015 ▸), *SHELXL2014* (Sheldrick, 2015 ▸), *DIAMOND* (Brandenburg & Putz, 2012 ▸) and *OLEX2* (Dolomanov *et al.*, 2009 ▸).

## supplementary crystallographic information

### Crystal data

**Table:**

Rb_2_O_2_·2NH_3_	*D*_x_ = 2.859 Mg m^−^^3^
*M**_r_* = 237.01	Mo *K*α radiation, λ = 0.71073 Å
Orthorhombic, *P**n**m**a*	Cell parameters from 1711 reflections
*a* = 7.3957 (7) Å	θ = 3.4–28.3°
*b* = 4.0932 (6) Å	µ = 17.66 mm^−^^1^
*c* = 18.1873 (17) Å	*T* = 123 K
*V* = 550.57 (11) Å^3^	Block, clear colourless
*Z* = 4	0.24 × 0.09 × 0.08 mm
*F*(000) = 440	

### Data collection

**Table:**

Agilent SuperNova Dual Source diffractometer with an Eos detector	641 independent reflections
Mirror monochromator	570 reflections with *I* > 2σ(*I*)
Detector resolution: 7.9851 pixels mm^-1^	*R*_int_ = 0.057
phi and ω scans	θ_max_ = 26.4°, θ_min_ = 3.6°
Absorption correction: analytical [CrysAlis PRO (Agilent, 2012), based on expressions derived by Clark & Reid (1995)]	*h* = −9→9
*T*_min_ = 0.064, *T*_max_ = 0.354	*k* = −5→4
2921 measured reflections	*l* = −22→22

### Refinement

**Table:**

Refinement on *F*^2^	0 restraints
Least-squares matrix: full	Hydrogen site location: difference Fourier map
*R*[*F*^2^ > 2σ(*F*^2^)] = 0.050	Only H-atom coordinates refined
*wR*(*F*^2^) = 0.118	*w* = 1/[σ^2^(*F*_o_^2^) + (0.0361*P*)^2^ + 7.6682*P*] where *P* = (*F*_o_^2^ + 2*F*_c_^2^)/3
*S* = 1.35	(Δ/σ)_max_ < 0.001
641 reflections	Δρ_max_ = 1.12 e Å^−^^3^
51 parameters	Δρ_min_ = −1.79 e Å^−^^3^

### Special details

**Table:**

Geometry. All esds (except the esd in the dihedral angle between two l.s. planes) are estimated using the full covariance matrix. The cell esds are taken into account individually in the estimation of esds in distances, angles and torsion angles; correlations between esds in cell parameters are only used when they are defined by crystal symmetry. An approximate (isotropic) treatment of cell esds is used for estimating esds involving l.s. planes.

### Fractional atomic coordinates and isotropic or equivalent isotropic displacement parameters (Å^2^)

**Table:**

	*x*	*y*	*z*	*U*_iso_*/*U*_eq_	Occ. (<1)
Rb1	0.11196 (14)	0.2500	0.42075 (5)	0.0164 (4)	
Rb2	−0.25614 (15)	1.2500	0.28803 (5)	0.0182 (4)	
O1	−0.1266 (11)	0.7500	0.3825 (4)	0.019 (2)	
O2	0.0133 (11)	0.7500	0.3206 (4)	0.022 (2)	
N2	0.6843 (16)	0.2500	0.4710 (6)	0.020 (3)	
N1	0.4060 (18)	0.650 (4)	0.3327 (7)	0.017 (4)	0.5
H1A	0.28 (2)	0.7500	0.340 (8)	0.025*	
H1B	0.42 (2)	0.7500	0.285 (8)	0.025*	
H1C	0.46 (2)	0.7500	0.364 (8)	0.025*	
H2A	0.58 (2)	0.2500	0.472 (8)	0.025*	
H2B	0.739 (13)	0.43 (3)	0.441 (5)	0.025*	

### Atomic displacement parameters (Å^2^)

**Table:**

	*U*^11^	*U*^22^	*U*^33^	*U*^12^	*U*^13^	*U*^23^
Rb1	0.0107 (5)	0.0268 (8)	0.0116 (5)	0.000	−0.0022 (4)	0.000
Rb2	0.0088 (5)	0.0345 (8)	0.0113 (5)	0.000	0.0003 (4)	0.000
O1	0.012 (4)	0.027 (6)	0.020 (4)	0.000	0.003 (3)	0.000
O2	0.011 (4)	0.040 (7)	0.014 (4)	0.000	0.000 (3)	0.000
N2	0.017 (5)	0.025 (7)	0.018 (5)	0.000	0.000 (4)	0.000
N1	0.016 (6)	0.019 (12)	0.016 (6)	−0.007 (6)	−0.002 (5)	0.000 (6)

### Geometric parameters (Å, º)

**Table:**

Rb1—Rb2^i^	3.6383 (15)	Rb2—N1^x^	3.507 (15)
Rb1—O1	2.790 (5)	Rb2—N1^xiii^	2.988 (14)
Rb1—O1^ii^	3.579 (8)	Rb2—N1^ix^	2.988 (14)
Rb1—O1^i^	2.790 (6)	O1—Rb1^ii^	3.579 (8)
Rb1—O2	2.836 (5)	O1—Rb1^viii^	2.790 (5)
Rb1—O2^i^	2.836 (5)	O1—Rb2^i^	2.839 (6)
Rb1—N2^iii^	3.292 (12)	O1—O2	1.530 (11)
Rb1—N2^iv^	3.215 (8)	O2—Rb1^viii^	2.836 (5)
Rb1—N2^v^	3.215 (8)	O2—Rb2^xiv^	3.316 (6)
Rb1—N2^vi^	3.215 (8)	O2—Rb2^xv^	3.316 (6)
Rb1—N1^vii^	3.157 (14)	O2—Rb2^i^	2.917 (6)
Rb1—N1	3.157 (14)	N2—Rb1^iv^	3.215 (8)
Rb2—O1	2.839 (6)	N2—Rb1^xvi^	3.292 (12)
Rb2—O1^viii^	2.839 (6)	N2—Rb1^xvii^	3.215 (8)
Rb2—O2^viii^	2.917 (6)	N2—Rb2^xviii^	3.356 (11)
Rb2—O2^ix^	3.316 (6)	N1—Rb1^viii^	3.652 (15)
Rb2—O2	2.917 (6)	N1—Rb2^xiv^	2.988 (14)
Rb2—O2^x^	3.316 (6)	N1—Rb2^xv^	3.507 (15)
Rb2—N2^xi^	3.356 (11)	N1—Rb2^xvi^	3.598 (14)
Rb2—N1^xi^	3.095 (15)	N1—Rb2^xviii^	3.095 (15)
Rb2—N1^xii^	3.095 (15)	N1—N1^xix^	0.82 (3)
			
O1^ii^—Rb1—Rb2^i^	133.30 (13)	O2—Rb2—N1^x^	53.2 (3)
O1^i^—Rb1—Rb2^i^	50.32 (13)	O2—Rb2—N1^xi^	150.4 (3)
O1—Rb1—Rb2^i^	50.32 (13)	O2—Rb2—N1^xii^	97.3 (3)
O1^i^—Rb1—O1^ii^	105.56 (17)	O2^ix^—Rb2—N1^x^	103.6 (3)
O1—Rb1—O1^i^	94.4 (2)	O2^viii^—Rb2—N1^ix^	59.4 (3)
O1—Rb1—O1^ii^	105.56 (17)	O2^viii^—Rb2—N1^x^	112.7 (3)
O1^i^—Rb1—O2	101.92 (19)	O2^viii^—Rb2—N1^xi^	97.3 (3)
O1^i^—Rb1—O2^i^	31.5 (2)	O2^x^—Rb2—N1^x^	51.0 (3)
O1—Rb1—O2	31.5 (2)	O2—Rb2—N1^xiii^	59.4 (3)
O1—Rb1—O2^i^	101.92 (19)	O2^viii^—Rb2—N1^xiii^	105.0 (3)
O1^i^—Rb1—N2^v^	156.3 (3)	O2—Rb2—N1^ix^	105.0 (3)
O1^i^—Rb1—N2^iii^	57.42 (17)	N2^xi^—Rb2—N1^x^	131.7 (3)
O1^i^—Rb1—N2^iv^	156.3 (3)	N1^xi^—Rb2—O2^x^	93.8 (3)
O1—Rb1—N2^v^	89.0 (2)	N1^xi^—Rb2—O2^ix^	54.2 (3)
O1—Rb1—N2^iv^	89.0 (2)	N1^ix^—Rb2—O2^ix^	55.3 (3)
O1—Rb1—N2^vi^	156.3 (3)	N1^xii^—Rb2—O2^ix^	93.8 (3)
O1—Rb1—N2^iii^	57.42 (17)	N1^xiii^—Rb2—O2^x^	55.3 (3)
O1^i^—Rb1—N2^vi^	88.98 (19)	N1^xii^—Rb2—O2^x^	54.2 (3)
O1—Rb1—N1	85.9 (3)	N1^xiii^—Rb2—O2^ix^	96.1 (3)
O1^i^—Rb1—N1^vii^	85.9 (3)	N1^ix^—Rb2—O2^x^	96.1 (3)
O1^i^—Rb1—N1	133.5 (3)	N1^xii^—Rb2—N2^xi^	68.5 (3)
O1—Rb1—N1^vii^	133.5 (3)	N1^xiii^—Rb2—N2^xi^	141.3 (3)
O2^i^—Rb1—Rb2^i^	51.77 (13)	N1^xi^—Rb2—N2^xi^	68.5 (3)
O2—Rb1—Rb2^i^	51.77 (13)	N1^ix^—Rb2—N2^xi^	141.3 (3)
O2—Rb1—O1^ii^	130.55 (13)	N1^xii^—Rb2—N1^xi^	63.8 (6)
O2^i^—Rb1—O1^ii^	130.55 (13)	N1^ix^—Rb2—N1^xi^	103.1 (5)
O2—Rb1—O2^i^	92.4 (2)	N1^ix^—Rb2—N1^xii^	143.7 (3)
O2—Rb1—N2^v^	93.14 (17)	N1^xiii^—Rb2—N1^x^	11.3 (4)
O2^i^—Rb1—N2^iv^	166.8 (2)	N1^xiii^—Rb2—N1^ix^	66.4 (6)
O2^i^—Rb1—N2^vi^	93.14 (17)	N1^ix^—Rb2—N1^x^	77.7 (3)
O2—Rb1—N2^iii^	86.0 (2)	N1^xiii^—Rb2—N1^xii^	103.1 (5)
O2—Rb1—N2^iv^	93.14 (17)	N1^xii^—Rb2—N1^x^	94.0 (2)
O2—Rb1—N2^vi^	166.8 (2)	N1^xiii^—Rb2—N1^xi^	143.7 (3)
O2^i^—Rb1—N2^v^	166.8 (2)	N1^xi^—Rb2—N1^x^	144.2 (4)
O2^i^—Rb1—N2^iii^	86.0 (2)	Rb1^viii^—O1—Rb1^ii^	74.44 (17)
O2—Rb1—N1	58.5 (3)	Rb1—O1—Rb1^viii^	94.4 (2)
O2—Rb1—N1^vii^	103.0 (3)	Rb1—O1—Rb1^ii^	74.44 (17)
O2^i^—Rb1—N1	103.0 (3)	Rb1—O1—Rb2^i^	80.53 (7)
O2^i^—Rb1—N1^vii^	58.5 (3)	Rb1^viii^—O1—Rb2	80.53 (7)
N2^iii^—Rb1—Rb2^i^	57.66 (19)	Rb1^viii^—O1—Rb2^i^	153.3 (3)
N2^vi^—Rb1—Rb2^i^	139.21 (14)	Rb1—O1—Rb2	153.3 (3)
N2^iv^—Rb1—Rb2^i^	139.21 (14)	Rb2—O1—Rb1^ii^	127.97 (15)
N2^v^—Rb1—Rb2^i^	139.21 (14)	Rb2^i^—O1—Rb1^ii^	127.97 (15)
N2^iv^—Rb1—O1^ii^	51.21 (19)	Rb2^i^—O1—Rb2	92.3 (2)
N2^v^—Rb1—O1^ii^	51.21 (19)	O2—O1—Rb1^ii^	135.7 (5)
N2^iii^—Rb1—O1^ii^	75.6 (2)	O2—O1—Rb1	75.9 (3)
N2^vi^—Rb1—O1^ii^	51.21 (19)	O2—O1—Rb1^viii^	75.9 (3)
N2^v^—Rb1—N2^iii^	106.3 (2)	O2—O1—Rb2	77.4 (3)
N2^vi^—Rb1—N2^iii^	106.3 (2)	O2—O1—Rb2^i^	77.4 (3)
N2^iv^—Rb1—N2^iii^	106.3 (2)	Rb1—O2—Rb1^viii^	92.4 (2)
N2^v^—Rb1—N2^vi^	79.1 (2)	Rb1^viii^—O2—Rb2	78.45 (8)
N2^v^—Rb1—N2^iv^	0.0 (5)	Rb1—O2—Rb2	144.3 (3)
N2^iv^—Rb1—N2^vi^	79.1 (2)	Rb1^viii^—O2—Rb2^xiv^	134.1 (3)
N1^vii^—Rb1—Rb2^i^	100.3 (2)	Rb1—O2—Rb2^xiv^	78.75 (10)
N1—Rb1—Rb2^i^	100.3 (2)	Rb1—O2—Rb2^i^	78.45 (8)
N1—Rb1—O1^ii^	119.1 (3)	Rb1—O2—Rb2^xv^	134.1 (3)
N1^vii^—Rb1—O1^ii^	119.1 (3)	Rb1^viii^—O2—Rb2^i^	144.3 (3)
N1—Rb1—N2^v^	70.0 (3)	Rb1^viii^—O2—Rb2^xv^	78.75 (10)
N1^vii^—Rb1—N2^vi^	70.0 (3)	Rb2^i^—O2—Rb2	89.1 (2)
N1—Rb1—N2^iv^	70.0 (3)	Rb2^xiv^—O2—Rb2^xv^	76.22 (17)
N1^vii^—Rb1—N2^iv^	108.5 (3)	Rb2—O2—Rb2^xv^	78.31 (10)
N1—Rb1—N2^vi^	108.5 (3)	Rb2^i^—O2—Rb2^xiv^	78.32 (10)
N1—Rb1—N2^iii^	143.4 (3)	Rb2—O2—Rb2^xiv^	131.6 (3)
N1^vii^—Rb1—N2^iii^	143.4 (3)	Rb2^i^—O2—Rb2^xv^	131.6 (3)
N1^vii^—Rb1—N2^v^	108.5 (3)	O1—O2—Rb1	72.6 (3)
N1—Rb1—N1^vii^	62.4 (6)	O1—O2—Rb1^viii^	72.6 (3)
O1—Rb2—O1^viii^	92.3 (2)	O1—O2—Rb2	71.8 (3)
O1^viii^—Rb2—O2^ix^	95.11 (15)	O1—O2—Rb2^xv^	141.86 (9)
O1—Rb2—O2^viii^	98.78 (18)	O1—O2—Rb2^xiv^	141.86 (9)
O1—Rb2—O2^x^	95.11 (15)	O1—O2—Rb2^i^	71.8 (3)
O1^viii^—Rb2—O2^x^	168.22 (18)	Rb1^iv^—N2—Rb1^xvi^	73.7 (2)
O1—Rb2—O2^ix^	168.22 (18)	Rb1^iv^—N2—Rb1^xvii^	79.1 (2)
O1^viii^—Rb2—O2^viii^	30.8 (2)	Rb1^xvii^—N2—Rb1^xvi^	73.7 (2)
O1^viii^—Rb2—O2	98.77 (18)	Rb1^xvi^—N2—Rb2^xviii^	66.4 (2)
O1—Rb2—O2	30.8 (2)	Rb1^xvii^—N2—Rb2^xviii^	123.1 (3)
O1—Rb2—N2^xi^	56.23 (17)	Rb1^iv^—N2—Rb2^xviii^	123.1 (3)
O1^viii^—Rb2—N2^xi^	56.23 (17)	Rb1—N1—Rb1^viii^	73.5 (3)
O1^viii^—Rb2—N1^ix^	85.1 (3)	Rb1—N1—Rb2^xvi^	161.2 (5)
O1—Rb2—N1^ix^	134.7 (3)	Rb1—N1—Rb2^xv^	116.4 (4)
O1—Rb2—N1^x^	76.0 (3)	Rb2^xiv^—N1—Rb1	79.2 (4)
O1—Rb2—N1^xi^	119.6 (3)	Rb2^xvi^—N1—Rb1^viii^	93.0 (4)
O1—Rb2—N1^xii^	74.5 (3)	Rb2^xv^—N1—Rb1^viii^	66.4 (3)
O1^viii^—Rb2—N1^xiii^	134.7 (3)	Rb2^xviii^—N1—Rb1	114.5 (5)
O1^viii^—Rb2—N1^xii^	119.6 (3)	Rb2^xiv^—N1—Rb1^viii^	116.9 (4)
O1^viii^—Rb2—N1^xi^	74.5 (3)	Rb2^xviii^—N1—Rb1^viii^	162.1 (5)
O1^viii^—Rb2—N1^x^	140.3 (3)	Rb2^xviii^—N1—Rb2^xvi^	75.0 (3)
O1—Rb2—N1^xiii^	85.1 (3)	Rb2^xviii^—N1—Rb2^xv^	118.8 (4)
O2^x^—Rb2—O2^ix^	76.22 (17)	Rb2^xv^—N1—Rb2^xvi^	67.5 (3)
O2—Rb2—O2^ix^	154.83 (12)	Rb2^xiv^—N1—Rb2^xv^	77.7 (3)
O2^viii^—Rb2—O2^ix^	92.24 (11)	Rb2^xiv^—N1—Rb2^xvi^	119.1 (4)
O2^viii^—Rb2—O2	89.1 (2)	Rb2^xiv^—N1—Rb2^xviii^	80.9 (4)
O2^viii^—Rb2—O2^x^	154.83 (12)	N1^xix^—N1—Rb1^viii^	47.7 (2)
O2—Rb2—O2^x^	92.24 (11)	N1^xix^—N1—Rb1	121.2 (3)
O2^viii^—Rb2—N2^xi^	83.6 (2)	N1^xix^—N1—Rb2^xv^	45.5 (2)
O2^ix^—Rb2—N2^xi^	121.53 (19)	N1^xix^—N1—Rb2^xvi^	46.9 (3)
O2^x^—Rb2—N2^xi^	121.53 (19)	N1^xix^—N1—Rb2^xiv^	123.2 (3)
O2—Rb2—N2^xi^	83.6 (2)	N1^xix^—N1—Rb2^xviii^	121.9 (3)
O2^viii^—Rb2—N1^xii^	150.4 (3)		

Symmetry codes: (i) *x*, *y*−1, *z*; (ii) −*x*, −*y*+1, −*z*+1; (iii) *x*−1, *y*, *z*; (iv) −*x*+1, −*y*+1, −*z*+1; (v) −*x*+1, *y*+1/2, −*z*+1; (vi) −*x*+1, *y*−1/2, −*z*+1; (vii) *x*, −*y*+1/2, *z*; (viii) *x*, *y*+1, *z*; (ix) *x*−1/2, *y*+1, −*z*+1/2; (x) *x*−1/2, *y*, −*z*+1/2; (xi) *x*−1, *y*+1, *z*; (xii) *x*−1, −*y*+3/2, *z*; (xiii) *x*−1/2, −*y*+3/2, −*z*+1/2; (xiv) *x*+1/2, *y*−1, −*z*+1/2; (xv) *x*+1/2, *y*, −*z*+1/2; (xvi) *x*+1, *y*, *z*; (xvii) −*x*+1, −*y*, −*z*+1; (xviii) *x*+1, *y*−1, *z*; (xix) *x*, −*y*+3/2, *z*.

### Hydrogen-bond geometry (Å, º)

**Table:**

*D*—H···*A*	*D*—H	H···*A*	*D*···*A*	*D*—H···*A*
N1—H1*A*···O2	1.05 (14)	1.98 (15)	2.941 (16)	151 (8)
N1—H1*B*···O2^xx^	0.97 (13)	2.04 (15)	2.926 (15)	151 (8)
N1—H1*C*···O1^xvi^	0.82 (14)	3.07 (16)	3.597 (16)	125 (11)
N2—H2*A*···N2^iv^	0.74 (16)	3.03 (12)	3.57 (2)	131 (6)
N2—H2*A*···N2^vi^	0.74 (16)	3.03 (12)	3.57 (2)	131 (6)
N2—H2*A*···N2^v^	0.74 (16)	3.03 (12)	3.57 (2)	131 (6)
N2—H2*B*···O1^xvi^	1.01 (11)	1.95 (11)	2.955 (10)	173 (8)

Symmetry codes: (iv) −*x*+1, −*y*+1, −*z*+1; (v) −*x*+1, *y*+1/2, −*z*+1; (vi) −*x*+1, *y*−1/2, −*z*+1; (xvi) *x*+1, *y*, *z*; (xx) *x*+1/2, −*y*+3/2, −*z*+1/2.
